# Size-Controlled and Optical Properties of Monodispersed Silver Nanoparticles Synthesized by the Radiolytic Reduction Method

**DOI:** 10.3390/ijms14047880

**Published:** 2013-04-11

**Authors:** Elias Saion, Elham Gharibshahi, Kazem Naghavi

**Affiliations:** 1 Physics Department, Universiti Putra Malaysia, UPM Serdang 43400, Selangor, Malaysia; E-Mail: elhamgs2002@yahoo.com; 2 Department of Science, Kerman branch, Islamic Azad University, Kerman 76351-31167, Iran; E-Mail: kazem.naghavi@gmail.com

**Keywords:** theory of metal nanoparticles, shell model, particle size, absorption maxima, conduction band, silver nanoparticles, radiolytic synthesis

## Abstract

Size-controlled and monodispersed silver nanoparticles were synthesized from an aqueous solution containing silver nitrate as a metal precursor, polyvinyl alcohol as a capping agent, isopropyl alcohol as hydrogen and hydroxyl radical scavengers, and deionized water as a solvent with a simple radiolytic method. The average particle size decreased with an increase in dose due to the domination of nucleation over ion association in the formation of the nanoparticles by gamma reduction. The silver nanoparticles exhibit a very sharp and strong absorption spectrum with the absorption maximum λ_max_ blue shifting with an increased dose, owing to a decrease in particle size. The absorption spectra of silver nanoparticles of various particle sizes were also calculated using a quantum physics treatment and an agreement was obtained with the experimental absorption data. The results suggest that the absorption spectrum of silver nanoparticles possibly derived from the intra-band excitations of conduction electrons from the lowest energy state (*n* = 5, *l* = 0) to higher energy states (*n* ≥ 6; Δ*l* = 0, ±1; Δ*s* = 0, ±1), allowed by the quantum numbers principle. This demonstrates that the absorption phenomenon of metal nanoparticles based on a quantum physics description could be exploited to be added into the fundamentals of metal nanoparticles and the related fields of nanoscience and nanotechnology.

## 1. Introduction

Ancient civilizations used metal nanoparticles for their brilliant colors, and they can be found in the stained glass windows of the Middle Ages, and yet, they continue to attract considerable attention today [1–3]. They exhibit size-dependent properties such as the tuning of absorption energy with particle size, a blue shift of absorption onset, and an enhancement of photocatalytic activities with a decrease in particle size. These novel properties are the consequence of a large number of surface atoms and three-dimensional quantum confinement of electrons, which are believed to be the factors regulating the physical and chemical properties of metal nanoparticles. However, a number of fundamental issues of metal nanoparticles have to be addressed. For instance, metal nanoparticles display a sharp absorption spectrum when illuminated with UV-visible light and, in this situation; they can perform as photocatalysts to boost a chemical reaction. Hence, it seems that the catalysts’ activity is directly linked to the action of conduction electrons, which provide the absorption spectrum of the metal nanoparticles. Because of their size in the nanoscale regime between the atomic and bulk structures, metal nanoparticles may serve as a model system that possesses both the quantum and classical physics principles, which may be used to describe their behaviors. Which of these principles could interpret accurately the behaviors of metal nanoparticles?

Among metallic nanoparticles, a noble silver (Ag) nanoparticle continues to be interesting nanomaterial in nanoscience owing to their excellent optical and electronic properties [4–6]. The research on this material has steered into many fields: catalysis [7], electrochemical sensors [8,9], antibacterial activity [10,11], degradation of environmental pollutants [12], biological labeling [13], drug delivery [14], surface-enhanced Raman scattering [15], and cancer therapy [16]. The optical properties of Ag nanoparticles are dictated by the geometrical parameters such as size and shape [17]. In recent years, much effort has been focused on the development of new strategies for the synthesis of Ag nanoparticles of high dispersion and uniform size and shape. They fall under two categories, *i.e.*, the chemical and physical approaches. The chemical approach utilizes several techniques such as the chemical, electrochemical, sol-gel, microemulsions, reverse micelles, and polyol methods [18–20]. To produce small clusters or aggregates of Ag nanoparticles, silver ions in aqueous solution were commonly reduced using a reducing agent, such as sodium borohydride [21], hydrazine [22], tetrabutylammonium borohydride [23], sodium citrate [24], dimethylformamide [25], ascorbic acid [26], and alcohols or polyols [27,28]. In the physical approach, the Ag ions in aqueous solution may be reduced by means of several irradiation techniques, including ultraviolet light [29,30], microwave [31], ultrasound [32], laser ablation [33], and gamma irradiation [34], without the need for a reducing agent.

Among the physical techniques, the synthesis of Ag nanoparticles using the radiolytic method has not been completely explored. Previous works emphasized only the mechanism of reduction and aggregation of Ag nanoparticles [35–38]. Nevertheless, this technique continues to receive considerable interest [39–42]. The method offers several advantages over the conventional methods: the process is simple and clean; controlled reduction of metal ions can be carried out without using a reducing agent or producing undesired oxidation products; it provides metal nanoparticles in a fully reduced, highly pure and highly stable state; it is harmless and environmentally friendly; it has proven to be a powerful method of synthesis at room temperature; and, it produces monodispersed nanoparticles [35–43]. The present study aims at examining the effect of dose on particle size of Ag nanoparticles and to interpret the optical properties with a new theory of metal nanoparticles based on quantum physics, which could add to the fundamental knowledge of metal nanoparticles and the related fields of nanoscience and nanotechnology.

## 2. Results and Discussion

### 2.1. Formation of Ag Nanoparticles in Colloidal PVA

The interaction of gamma photons with matter involves several distinct processes depending on the energy of the photons and on the density and atomic number of the medium. 1.25-MeV ^60^Co gamma rays interact with matter in aqueous solution by photoelectric absorption, Compton scattering, and pair production, resulting in the formation of secondary electrons, which is mostly coming from the Compton scattering effect. These free and energetic electrons can induce several reactive species such as hydrated electrons (e^−^_aq_), hydroxyl radicals (OH^•^), and hydrogen radicals (H^•^) by radiolysis of water (Reaction 1).

(1) $${\text{H}}_{2}\text{O}\overset{\gamma -\text{rays}}{\to }{\text{e}}_{\text{aq}}^{-},{\text{H}}_{3}\text{O},{\text{H}}^{•},{\text{OH}}^{•},{\text{H}}_{2},{\text{H}}_{2}{\text{O}}_{2},{\text{H}}_{3}{\text{O}}^{+}\;\;\;(\text{radiolysis of water})$$

The formation of Ag nanoparticles by gamma radiolytic method can be divided into two steps. First occurs the formation of the atom by the nucleation process; this is followed by the formation of nanoparticles by the aggregation process. The nucleation process can be described by the following reactions. The hydrated electrons (e^−^_aq_) are a strong reducing agent and can reduce Ag ions (Ag^+^) into zero-valent Ag atoms (Ag^0^) (reaction 2) [35]. In our case, the Ag ions, Ag^+^ come from silver nitrate in an aqueous solution of PVA used as a stabilizing agent to restrict the agglomeration of reduced Ag atoms, thus limiting the aggregation and the size of Ag nanoparticles.

(2) $${\text{Ag}}^{+}+{\text{e}}_{\text{aq}}^{-}\to {\text{Ag}}^{0}\;\;\;(\text{nucleation})$$

The H^•^ and OH^•^ radicals formed in radiolysis of water, (Reaction 1) are also strong reducing agents in aqueous solution. To avoid this oxidation, the radicals were scavenged by adding isopropanol (IPA). The H^•^ and OH^•^ radicals can react with IPA molecules during and after irradiation to form isopropanol radicals (IPA^•^) (Reactions 3 and 4), which then react with Ag ions, Ag^+^ to form zero-valent metal atoms, Ag^0^ (Reaction 5). However, the reduction of Ag ions, Ag^+^ by hydrated electrons is the main process for the formation of Ag nanoparticles under gamma irradiation [44].

(3) $${\text{H}}^{•}+{\text{CH}}_{3}-\text{CH}(\text{OH}){-\text{CH}}_{3}\to {\text{CH}}_{3}{-\text{C}}^{•}\;(\text{OH}){-\text{CH}}_{3}+{\text{H}}_{2}\;\;\;(\text{radicals formation})$$

(4) $${\text{OH}}^{•}+{\text{CH}}_{3}-\text{CH}(\text{OH}){-\text{CH}}_{3}\to {\text{CH}}_{3}{-\text{C}}^{•}\;(\text{OH}){-\text{CH}}_{3}+{\text{H}}_{2}\text{O }\;\;(\text{radicals formation})$$

(5) $${\text{Ag}}^{+}+{\text{CH}}_{3}{-\text{C}}^{•}\;(\text{OH}){-\text{CH}}_{3}\to {\text{CH}}_{3}{-\text{CO}-\text{CH}}_{3}+{\text{H}}^{+}+{\text{Ag}}^{0}\;\;\;(\text{nucleation})$$

The formation of Ag nanoparticles by the aggregation process can be described by the following reactions. The zero-valent Ag^0^ finally coalesce to form aggregates Ag^0^_m+1_ (Reaction 6). The agglomerated Ag nanoparticles, Ag^0^_m+1_ can also combine with Ag^+^ to form larger Ag ions, Ag^+^_m+2_ (Reaction 7), which can then be reduced by hydrated electrons into larger Ag nanoparticles, (Ag^0^_m+2_) (Reaction 8). The binding energy between two Ag atoms is stronger than the Ag–PVA bond energy [45]. Hence, when the Ag atoms stumble upon each other, they are attracted and diffused into a larger Ag nanocluster, Ag^0^_m+n_ (Reaction 9).

(6) $${{\text{Ag}}^{0}}_{\text{m}}\;+\;{\text{Ag}}^{0}\to {{\text{Ag}}^{0}}_{\text{m}+1}\;\;\;(\text{agglomeration})$$

(7) $${{\text{Ag}}^{0}}_{\text{m}+1}\;+\;{\text{Ag}}^{+}\to {{\text{Ag}}^{+}}_{\text{m}+2}\;\;\;(\text{ion association})$$

(8) $${{\text{Ag}}^{+}}_{\text{m}+2}\;+\;{\text{e}}_{\text{aq}}^{-}\to {{\text{Ag}}^{0}}_{\text{m}+2}\;\;\;(\text{agglomeration})$$

(9) $${{\text{Ag}}^{0}}_{\text{m}}\;+\;{{\text{Ag}}^{0}}_{\text{n}}\to {{\text{Ag}}^{0}}_{\text{m}+\text{n}}\;\;\;(\text{agglomeration})$$

To prevent an increase in cluster size, a polymer is commonly used. The polymer is adsorbed on the clusters’ surface to reduce the surface tension [46]. In this experiment, PVA was adopted. In aqueous medium, PVA radicals (PVA^•^) might also be formed by PVA reacting with OH^•^ radicals that were not scavenged by IPA. However, the PVA^•^ then react with each other to initiate an intra- or inter-molecular crosslinking between PVA chains, resulting in a three-dimensional PVA network (Reaction 10) [47], which encircles the Ag nanoparticles [48]. The adsorption of PVA network on the surface of Ag nanoparticles can reduce the surface energy and decrease further the agglomeration process of Ag nanoparticles [49]. This increases the degree of polymer capping on the surface of nanoparticles leading to smaller particle sizes.

(10) $${\text{PVA}}^{•}+{\text{PVA}}^{•}\to \text{PVA}-\text{PVA }\;\;(\text{crosslinked polymer})$$

**Figure 1:**
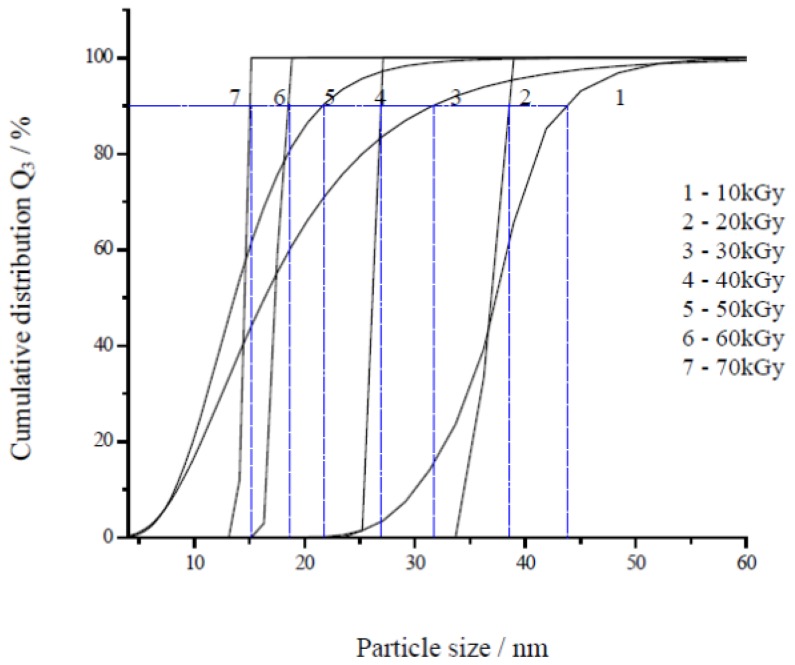
Cumulative distribution of particle size of monodispersed Ag nanoparticles synthesized from AgNO_3_ concentration of 4.2 × 10^−4^ M in 5 wt% PVA at different absorbed doses of (1) 10, (2) 20, (3) 30, (4) 40, (5) 50, (6) 60, and (7) 70 kGy.

**Figure 2:**
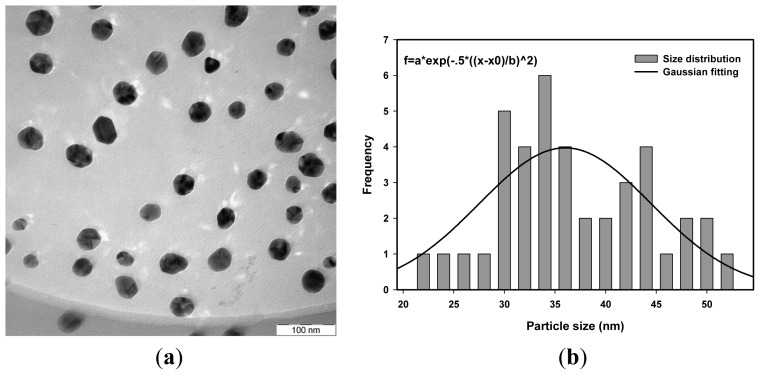
(**a**) TEM images and (**b**) TEM size distribution and Gaussian fitting of monodispersed Ag nanoparticles synthesized from AgNO_3_ concentration of 4.2 × 10^−4^ M in 5 wt% PVA and irradiated at 20 kGy.

**Figure 3:**
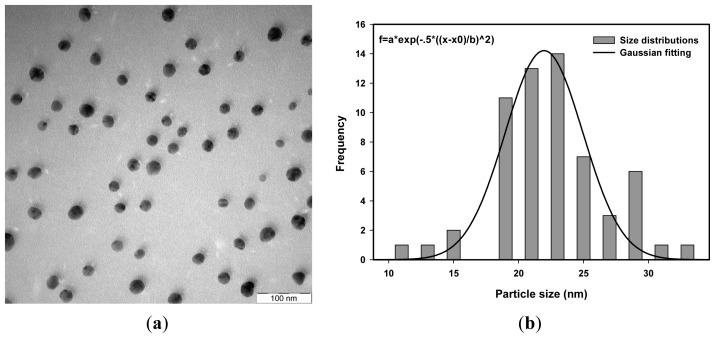
(**a**) TEM images and (**b**) TEM size distribution and Gaussian fitting of monodispersed Ag nanoparticles synthesized from AgNO_3_ concentration of 4.2 × 10^−4^ M in 5 wt% PVA and irradiated at 40 kGy.

**Figure 4:**
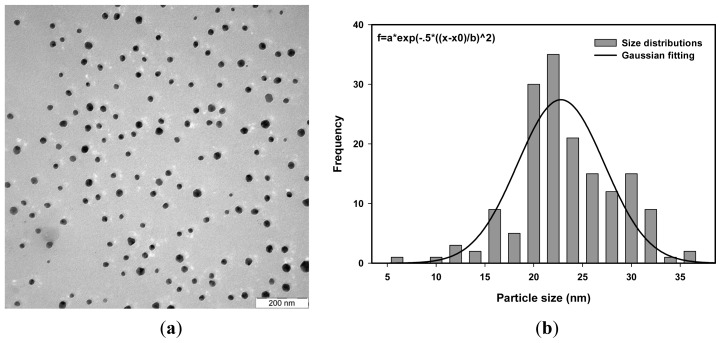
(**a**) TEM images and (**b**) TEM size distribution and Gaussian fitting of monodispersed Ag nanoparticles synthesized from AgNO_3_ concentration of 4.2 × 10^−4^ M in 5 wt% PVA and irradiated at 50 kGy.

The average particle size of Ag nanoparticles was determined from the photon cross correlation spectroscopy (PCCS) measurements using the cumulative distribution at 90% [50] as shown in Figure 1. The PCCS distribution depends on the average size of the nanoparticles. It gives a short tailing effect at low percentage of cumulative distribution for a narrow particle size distribution and a long tailing effect for a wide particle size distribution. Figures 2a, 3a and 4a show the transmission electron microscopy (TEM) images of Ag nanoparticles of 4.2 × 10^−4^ M Ag ions concentration and irradiated at doses of 20, 40 and 50 kGy, respectively. One of the TEM images, Figure 2a, was purposely measured at high magnification to demonstrate the monodispersed Ag nanoparticles obtained using the radiolytic reduction method. The TEM images and TEM size distribution show a well-monodispersed spherical shape in the size range of 22–52 nm, 14–32 and 10–36 nm for 20, 40 and 50 kGy doses, respectively. The TEM size distributions in Figures 2b, 3b and 4b were obtained and the average particle sizes were determined from the histograms to be 36.0, 25.0 and 22.5 nm for 20, 40 and 50 kGy, respectively. The discussion on the size width that decreased with increasing dose has been debated recently [51]. The average particle sizes synthesized at doses of 20, 40, and 50 kGy were comparable with the PCCS measurements as shown in Table 1 This suggests that a simple PCCS technique is applicable to determine particle size of colloidal Ag nanoparticles in the present size range.

### 2.2. Effect of Dose

**Figure 5:**
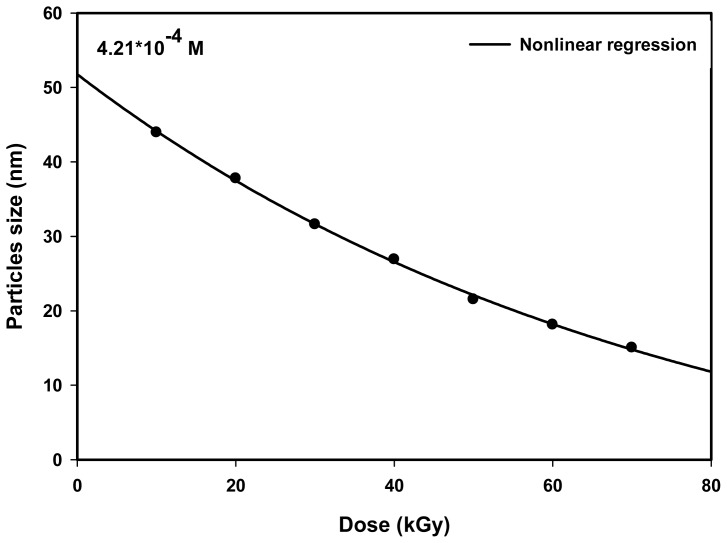
Average particle size *versus* dose for Ag nanoparticles synthesized from AgNO_3_ concentrations of 4.2 × 10^−4^ and 5 wt% PVA, showing exponential relationship between particle size and dose with dose sensitivity parameters *D*_0_ of 74.59 kGy.

Figure 5 shows the particles size *versus* the dose for Ag nanoparticles synthesized by radiolytic method. There is a competition between the nucleation and growth processes to make up Ag nanoparticles. The general trend is that the average particle size decreased with an increasing dose. At high doses, where the number of nucleation events is more than the number of unreduced ions, the radiolytic reduction synthesis produces smaller particle sizes. At low doses, however, where the nucleation concentration is less than the concentration of unreduced ions, the zero-valent agglomerated atoms can be ionized by unreduced ions and later reduced by hydrate electrons to produce even larger Ag nanoparticles (Reactions 6 and 7) [39]. The figures illustrate that the largest particle size is seen at the lowest dose of 10 kGy, and the smallest particle size is seen at the highest dose of 70 kGy. The average particle sizes were found to be 43.9 and 15.0 nm for 10 and 70 kGy, respectively. We expect that by increasing the precursor concentration, the particle size would become larger due to higher metal ions concentration, thus allowing more nucleation and aggregation processes to take place in the formation of Ag nanoparticles.

The particle size decreasing exponentially with the increasing dose may be fitted to an empirical relation of the form:

(11) $$d=d_{\text{max}}+B\text{exp}\left(-D/D_{0}\right)$$

where *d* is the average diameter of the nanoparticle at dose *D; d*_max_ is the nanoparticle diameter at the highest dose of 70 kGy; *d*_0_ is the nanoparticle diameter at the lowest dose of 10 kGy; *B* is the difference between *d*_max_ and *d*_0_; and *D*_0_ is the dose sensitivity parameter for particle size formation at a given precursor concentration. The values of *D*_0_ can be determined from the inverse of the gradient of ln[(*d* − *d*_max_)/B] *versus D*. The dose sensitivity parameters *D*_0_were found to be 74.63 kGy.

### 2.3. Optical Properties

**Figure 6:**
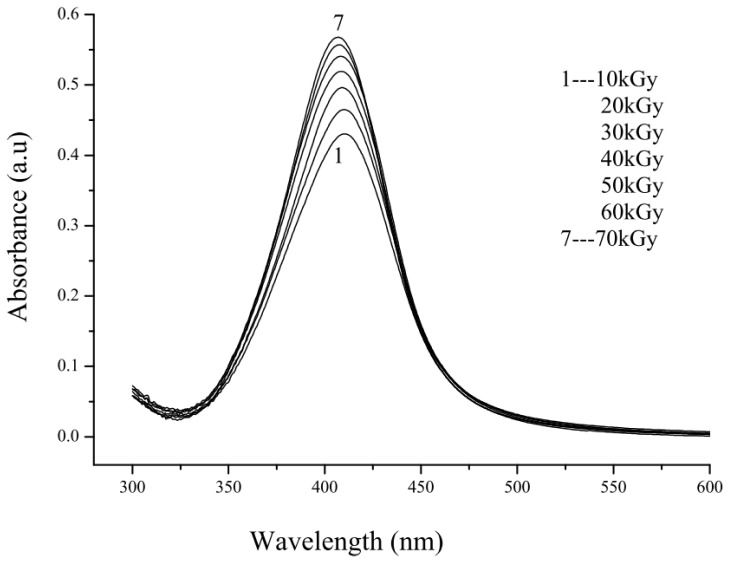
Sharp absorption spectra of Ag nanoparticles synthesized from 4.2 × 10^−4^ M AgNO_3_ concentration and 5 wt% PVA at different doses of 10 to 70 kGy. The absorption maximum λ_max_ blue shifted toward lower wavelength with increasing dose owing to decreasing particle size.

The evolution of absorption spectrum of Ag nanoparticles synthesized from 4.2 × 10^−4^ M precursor concentration and 5 wt% PVA with increasing dose from 10 to 70 kGy is shown in Figure 6. The absorbance increased with increasing dose owing to the number of Ag nanoparticles multiplied with increasing dose. The color of the colloidal solution changes from yellow to dark yellow on increasing dose due to increase in the numbers of Ag^+^ ions that have been reduced to zero-valent Ag_0_ atoms. This means that the number of Ag nanoparticles of smaller sizes increased with an increasing dose. The absorption peak or absorption maximum λ_max_ blue shifted toward lower wavelength with increasing dose, indicating the particle size decreases as the dose increases. This is quite easy to explain when the conduction electrons that produced the absorption spectrum could be treated using a quantum physics concept.

The absorption spectrum of metal nanoparticles is a well-documented phenomenon of classical physics since 1908, when the theory of particle scattering and absorbing light was first introduced by Gustav Mie [35]. It has successfully described analytically the absorption spectrum of metal nanoparticles in terms of the localized surface Plasmon resonance (LSPR), *i.e.*, the coherent oscillation of conduction electrons on the surface of metal nanoparticles in resonance with the electromagnetic waves at the metal–dielectric interface [52–55]. For a technical understanding, however, perhaps it should be left to the experts to scrutinize the complex physics and mathematics for obtaining and interpreting the theoretical data. In quantum physics, the absorption spectrum of metal nanoparticles may be described as a result of the intra-band excitations of conduction electrons from the lowest energy state to higher energy states within the conduction band of metal nanoparticles [56,57].

Recently, we have demonstrated that the absorption maxima λ_max_ of platinum (Pt) nanoparticles calculated using a new theory of metal nanoparticles agreed very well with the measured absorption maxima λ_max_ of radiolytic synthesized Pt nanoparticles at about 215 and 260 nm [58]. The theory is based on two simple assumptions: that metal nanoparticles have both geometric and electronic structures, which are both thermodynamically stable. The geometric structure may take a solid metal sphere built up from the primitive unit cells face-centered-cubic (FCC) lattice structure, and the electronic structure may follow the Jellium shell model of their respective atom. For an example, a Pt atom with the electronic configuration of Pt(78): (Xe) (5d^9^) (6s^1^) should produce a Pt nanoparticle with the electronic configuration of Pt(78)_N_: (Xe)_N_ (5d^9^)_N_ (6s^1^)_N_, where N is the number of atoms in the nanoparticle. Using the quantum mechanics principle of quantum numbers, we have identified that the absorption peaks of 215 and 260 nm were derived from the intra-band excitations of conduction electrons from the lowest energy states of quantum numbers (*n* = 5, *l* = 2) and (*n* = 6, *l* = 0) to higher energy states of quantum numbers (*n* ≥ 6; Δ*l* = 0, ±1; Δ*s* = 0, ±1) and (*n* ≥ 7; Δ*l* = 0, ±1; Δ*s* = 0, ±1), respectively.

According to this model, the Ag nanoparticle can be considered an isolated solid metal sphere of diameter *d* comprising of *N* number of atoms that are confined in the FCC lattice structure. In solid state physics, the band theory of metals considers that outer electronic orbitals of participated atoms of nanoparticles can overlap into a conduction band occupied by conduction electrons. The electronic configuration of Ag nanoparticles can be categorized in spectroscopy notation as Ag(47)_N_: (Kr)_N_ (4d^10^)_N_(5s^1^)_N_, which is based on the Ag atom electronic configuration Ag(47): (Kr) (4d^10^) (5s^1^). For the Ag nanoparticle, the conduction electrons are represented by (5s^1^)_N_ valence electrons and they are considered not entirely free—contradicted by the plasmonic description—but weakly bound to the crystal backbone at the lowest energy state of quantum numbers (*n* = 5, *l* = 0). When electromagnetic plane wave stricks Ag nanoparticle, the conduction electrons excite to higher energy states of quantum numbers (*n* ≥ 6; Δ*l* = 0, ±1; Δ*s* = 0, ±1), allowed by the quantum numbers principle.

The density energy functional of conduction electrons may be taken from the Thomas–Fermi–Dirac–Weizsacker model, which is the density functional theory fundamental, where the ground state electron density ρ(***r***) is the basic variable for all ground state properties [59–64]. The Euler–Lagrangian equation *E*[ρ(***r***)] can be represented as

(12) $$\frac{5}{3}C_{k}\int \rho {\left(r\right)}^{2/3}dr+\frac{\eta }{8}\left[\frac{{\left|\nabla \rho (r)\right|}^{2}}{\rho^{2}(r)}-2\frac{\nabla^{2}\rho (r)}{\rho (r)}\right]+v(r)+\int \frac{\sigma (r^{\prime })}{\left|r-r^{\prime }\right|}dr^{\prime }-\frac{4}{3}C_{e}\int \rho {\left(r\right)}^{1/3}dr=E_{0}$$

where, ρ(***r***) is the density of conduction electrons of Ag nanoparticle, *E*_0_ is the Fermi energy, *r* is the displacement of conduction electrons from the center of the sphere, which is dependent on Bohr radius *a*_0_, atomic number *Z*, and quantum numbers *n*, *l*, and, *s*. The first term is the Thomas–Fermi kinetic energy of homogeneous free electron gas system with *C_k_* as a constant [59,60]. The second term is the Weizsacker correction for modifying the Thomas–Fermi kinetic energy by inclusion of the exchange and correlation energy terms of inhomogeneous electron density with η as a constant [64]. The third term is the potential energy of the system. The fourth term is the classical Coulomb potential energy of electron–electron interactions. The final term is the non-classical exchange-correlation energy containing all the remaining quantum effects not captured by the kinetic energy; the classical Coulomb potential with *C_e_* is the Thomas–Fermi–Dirac non-classical exchange-correlation energy constant [63]. Details of the quantum mechanical treatment can be found in our earlier publications [56,57]. It can be shown that the relation between the density and absorption may be written as ρ ≈ (*Z*/σ)^3/2^, where *Z* is the atomic number. The transformation of density energy functional *E*[ρ(*r*)] into absorption energy functional *E*[σ(*r*)] can be calculated with a relatively simple mathematics involving only the integration and differentiation. The second order differential equation of the final absorption energy functional may be solved numerically [56,57]. All possible intra-band electronic transitions allowed would produce the same absorption maximum for a given diameter since the energy states of quantum numbers *n* ≥ 6 near the Fermi level are very close to or overlap each other [56,57]. Other parameters required for the calculation of absorption spectrum of Ag nanoparticles are simply the particle size, atomic number (*Z* = 47), Fermi energy (*E*_0_ = 5.49 eV) and lattice constant (0.408).

**Figure 7:**
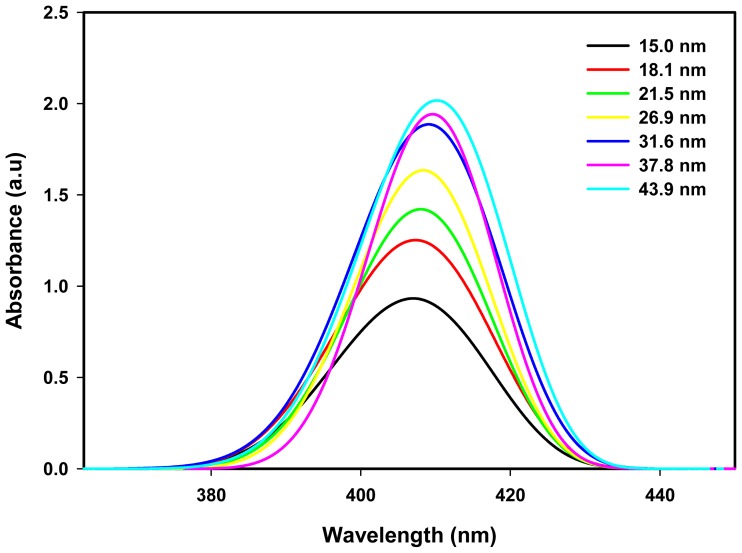
Theoretical absorption spectra of Ag nanoparticles for various diameters from 15.0 to 43.9 nm imitating the particle sizes obtained from the experiment at doses of 10 to 70 kGy. The absorption spectra are attributed to the intra-band electronic excitations of conduction electrons from the lowest energy state of quantum numbers (*n* = 5; *l* = 0) to higher energy states of quantum numbers (*n* ≥ 6; Δ*l* = 0, ±1; Δ*s* = 0, ±1) allowed by the quantum numbers principle.

Figure 7 show the calculated absorption spectrum of Ag nanoparticles of various diameters from 15.0 to 43.9 nm imitating the measured absorption spectrum of the same diameters obtained at doses of 10 to 70 kGy. Here, for each particle size, the calculated absorption spectrum is represented by one of the possible intra-band electronic transitions. It is clear that the theoretical absorption spectrum (Figure 7) and the experimental absorption spectrum (Figure 6) are not similar in terms of the height of the maximum and the width of the peak. The reason is that the calculated spectrum is based on a single Ag nanoparticle at a given diameter, while the measured spectrum was obtained from many Ag nanoparticles of the same average diameter. Figure 7 show the height of the maximum and the width of the peak of the calculated spectrum increase with increasing particle size and also the absorption maximum red shifted with increasing size. For the larger particle sizes, the number of atoms required to make up the nanoparticles is enormous, so the number of conduction electrons used in the computation is more than those of smaller particle sizes and this increases the height of the maximum and the width of the peak. The most important information between the theoretical and experimental results is that the simulated absorption maximum, λ_max_ consistent with the measured values as shown in Table 2. The results indicate that the absorption spectrum of Ag nanoparticles (Figure 6) most likely originated from the intra-band electronic excitations of conduction electrons from the lowest energy state of quantum numbers (*n* = 5; *l* = 0) to higher energy states of quantum numbers (*n* ≥ 6; Δ*l* = 0, ±1; Δ*s* = 0, ±1) within the conduction band of Ag nanoparticles.

**Figure 8:**
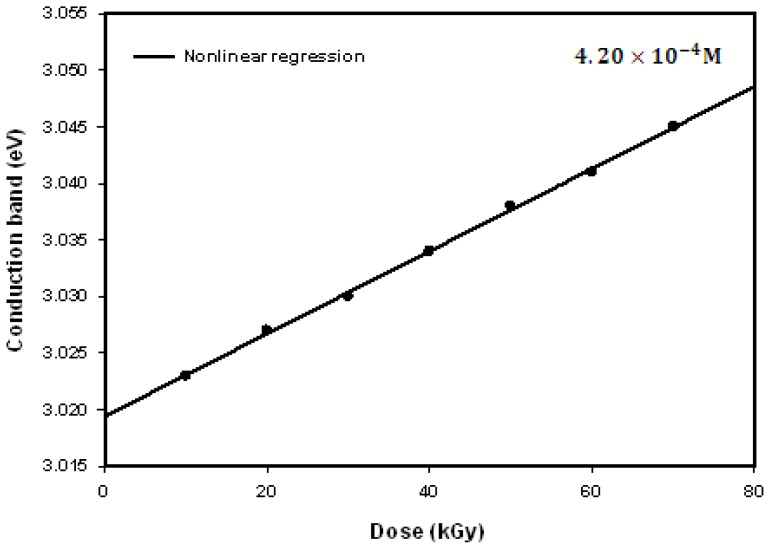
Conduction band of Ag nanoparticles increased nonlinearly with increasing dose owing to decreasing particle size as a result of the domination of nucleation process over ion association in the formation of nanoparticles at higher doses.

The conduction band energy, *E* of Ag nanoparticles may be calculated according to *E*=*hc*/λ_max_, where *h* is the Planck’s constant, *c* the speed of light, and λ_max_ the wavelength of the absorption maxima. The conduction band energy represents the amount of energy required to excite the conduction electrons from the lowest energy state to higher energy states influenced by the UV-visible electromagnetic radiation. Figure 8 shows the nonlinear relationship between the conduction band and the dose. The absorption maximum λ_max_ blue shifted toward lower wavelength from 410 to 407 nm with increasing dose, corresponding to conduction band of 3.023 to 3.045 eV, respectively. This is the result of decreasing particle size from 43.9 nm at 10 kGy to 15.0 nm at 70 kGy. For smaller particles, the number of atoms to make up the nanoparticles is few, so that conduction electrons are less attracted to protons of the Ag nanoparticles and, consequently increase the conduction band and decrease the absorption peak of Ag nanoparticles.

**Figure 9:**
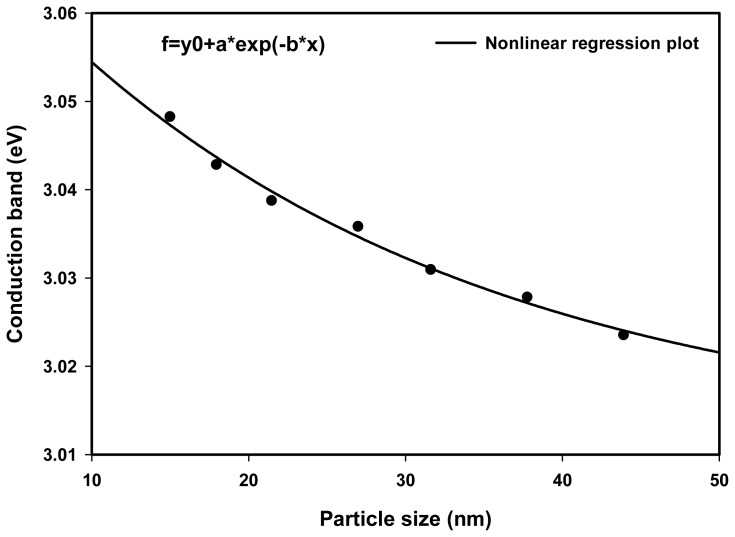
Conduction band of Ag nanoparticles decreased nonlinearly with increasing particle size, exclusively known as the quantum confinement effect, owing to the conduction band electrons that are more attracted to the crystal backbone of larger particle sizes.

Figure 9 shows that the conduction band of Ag nanoparticles decreased nonlinearly with decreasing particle size known as the quantum confinement effect of electrons. This is because, for larger particle sizes, the number of atoms comprising the nanoparticles is enormous and its conduction band electrons are more attracted to the crystal backbone of Ag nanoparticles when compared to smaller particle sizes, thus reducing the conduction band energy with increasing particle size.

## 3. Experimental Section

Silver nitrate, AgNO_3_ (99%), was used as a precursor, polyvinyl alcohol (PVA) (MW 10,000) as a capping agent, isopropanol as a radical scavenger of hydroxyl radicals, and deionized water as a solvent. All the chemical reagents were of research grade supplied by Sigma–Aldrich, St. Louis, Missouri, USA and used without further purification. The 5-wt% PVA stock solution was made by dissolving 14.0 g PVA powder in 280 mL deionized water at 90 °C. The solution was magnetically stirred for 3 h and was bubbled with nitrogen gas (99.5%) in order to remove oxygen. The Ag ions concentration of 4.2 × 10^−4^ M together with 1 mL isopropanol was added into PVA solution and stirred for 2 h. The sample was prepared and stored in a dark room to avoid reduction by light. The bulk sample was divided into seven parts and each irradiated at different doses of 10 to 70 kGy using 1.25-MeV ^60^Co γ-rays source. The radiation-induced synthesis offers additional benefits over the other conventional methods because it produces fully reduced and highly pure Ag nanoparticles free of byproducts or reducing agents.

Nanophox based on PCCS (Sympatec GmbH, D-38678 Clausthal-Zellerfeld, Germany) was used throughout this work to determine the particle size of prepared colloid Ag nanoparticles. The particle size and size distribution of some samples were also determined from TEM micrograph (Hitachi, H-7500, Tokyo, Japan). The TEM characterization was carried out at 100 kV. The optical properties of silver nanoparticles were characterized using UV-visible absorption spectroscopy (UV-1650PC Shimadzu, Kyoto, Japan). In the actual UV-visible absorption measurements, the monochromatic beam was split into two equal intensity beams. One beam, the sample, passed through a transparent cuvette containing the irradiated sample (AgNO_3_, PVA, IPA, and water); the other beam, the reference, passed through an identical cuvette containing irradiated reference sample (PVA, IPA, and water). All samples were diluted at the same dilution for the purpose of UV-visible absorption measurements. Prior to actual sample measurement, both cuvettes were filled with the irradiated sample and the irradiated reference sample to define the base line of the spectrophotometer system. This procedure is a necessity to ensure any absorption peaks produced from other radiochemical reactions would be discounted by the spectrophotometer system.

## 4. Conclusions

Silver nanoparticles stabilized in aqueous PVA solution were synthesized from a reduction of silver nitrate by the gamma radiolytic method. The average particle size decreased exponentially with an increasing dose and fitted to an empirical relation with the dose sensitivity parameters of 74.59 kGy. The absorption maximum at 410-nm absorption spectrum shifted to lower wavelength with increasing dose owing to a decrease in particle size at higher doses. Consequently, the conduction band energy derived from the absorption minimum increased with increasing particle size owing to the quantum confinement effect. The absorption spectrum of Ag nanoparticles was calculated using a spherical shell model and quantum physics principles and found that the calculated absorption maximum values are consistent with the measured results for the synthesized Ag nanoparticles. The results validate that the absorption peak of Ag nanoparticles is most probably derived from the intra-band excitations of conduction electrons from the lowest energy state of quantum numbers (*n* = 5, *l* = 0) to higher energy states of quantum numbers (*n* ≥ 6; Δ*l* = 0, ±1; Δ*s* = 0, ±1) allowed by the quantum numbers principle. This demonstrates that the absorption spectrum of metal nanoparticles based on quantum physics can be exploited further, which can be added to the fundamental understanding of metal nanoparticles and the related fields of nanoscience and nanotechnology.

## Figures and Tables

**Table 1:** Comparison of average particle sizes of Ag nanoparticles measured by TEM and PCCS methods for the particles synthesized from 4.2 × 10^−4^ M AgNO_3_ in 5 wt% PVA and irradiated with 20, 40, and 50 kGy.

Dose (kGy)	Average size measurement using TEM (nm)	Average size measurement using PCCS (nm)
20	36.0	37.8
40	25.0	26.9
50	22.5	21.5

**Table 2:** Average particle size, absorption maximum, and conduction band energy of Ag nanoparticles synthesized by radiolytic reduction method at doses of 10 to 70 kGy.

Dose (kGy)	Particle size (nm)	Experiment λ_max_ (nm)	Theory λ_max_ (nm)	Conduction band (eV)
10	43.9	410.0	410.12	3.023
20	37.8	409.5	409.54	3.027
30	31.6	409.0	409.12	3.031
40	26.9	408.5	408.46	3.034
50	21.5	408.0	408.07	3.038
60	18.1	407.5	407.52	3.041
70	15.0	407.0	406.80	3.045
